# Nonswelling and Hydrolytically Stable Hydrogels Uncover Cellular Mechanosensing in 3D

**DOI:** 10.1002/advs.202105325

**Published:** 2022-02-20

**Authors:** Hongyan Long, Bart E. Vos, Timo Betz, Brendon M. Baker, Britta Trappmann

**Affiliations:** ^1^ Bioactive Materials Laboratory Max Planck Institute for Molecular Biomedicine Röntgenstraße 20 Münster 48149 Germany; ^2^ Third Institute of Physics – Biophysics Georg August University Göttingen Göttingen 37077 Germany; ^3^ Engineered Microenvironments and Mechanobiology Lab Department of Biomedical Engineering University of Michigan Ann Arbor MI 48109 USA

**Keywords:** 3D cell spreading, cellular mechanosensing, matrix degradability, matrix stiffness, synthetic hydrogels

## Abstract

While matrix stiffness regulates cell behavior on 2D substrates, recent studies using synthetic hydrogels have suggested that in 3D environments, cell behavior is primarily impacted by matrix degradability, independent of stiffness. However, these studies did not consider the potential impact of other confounding matrix parameters that typically covary with changes in stiffness, particularly, hydrogel swelling and hydrolytic stability, which may explain the previously observed distinctions in cell response in 2D versus 3D settings. To investigate how cells sense matrix stiffness in 3D environments, a nonswelling, hydrolytically stable, linearly elastic synthetic hydrogel model is developed in which matrix stiffness and degradability can be tuned independently. It is found that matrix degradability regulates cell spreading kinetics, while matrix stiffness dictates the final spread area once cells achieve equilibrium spreading. Importantly, the differentiation of human mesenchymal stromal cells toward adipocytes or osteoblasts is regulated by the spread state of progenitor cells upon initiating differentiation. These studies uncover matrix stiffness as a major regulator of cell function not just in 2D, but also in 3D environments, and identify matrix degradability as a critical microenvironmental feature in 3D that in conjunction with matrix stiffness dictates cell spreading, cytoskeletal state, and stem cell differentiation outcomes.

## Graphical abstract

This study establishes a linearly elastic, 3D hydrogel cell culture model, in which matrix stiffness, adhesiveness, and degradability can be independently tuned without concurrent changes in other hydrogel properties, in particular swelling and hydrolytic stability. Using this model, the authors determine that matrix degradability regulates cell spreading kinetics, while matrix stiffness dictates the final spread state once cells achieve equilibrium spreading.

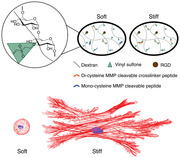

## 1 Introduction

Adhesive interactions between cells and their surrounding extracellular matrix (ECM) regulate many basic cellular functions,^[^ ^1^ ^]^ such as spreading,^[^ ^2^ ^]^ migration,^[^ ^3^ ^]^ proliferation,^[^ ^4^ ^]^ or stem cell differentiation.^[^ ^5^ ^]^ Thus, understanding these interactions is critical for the design of novel materials for tissue engineering applications.^[^ ^6^ ^]^ Synthetic hydrogels with independently tunable biochemical and mechanical properties have been instrumental in extending our understanding of how individual ECM properties impact cell behavior.^[^ ^6^, ^7^ ^]^ In particular, matrix stiffness has emerged as a major regulator of the behavior of cells cultured atop elastic hydrogels,^[^ ^8^ ^]^ where increasing substrate stiffness enhances cell spreading, actin stress fiber formation, proliferation and human mesenchymal stromal cell (hMSC) differentiation toward an osteogenic lineage.^[^ ^2^, ^5^, ^9^ ^]^ However, if and how matrix stiffness regulates cell fate and function in more physiological, 3D environments is not well‐established.^[^ ^10^ ^]^ While some studies have confirmed the importance of matrix stiffness in 3D,^[^ ^11^ ^]^ others have shown that in physically confined environments, cell function is only regulated by matrix degradability, independent of matrix stiffness.^[^ ^12^ ^]^ This discrepancy has led to the overall notion that 2D models may not recapitulate the ECM stiffness response in 3D models and by extension, physiological tissues. However, the majority of 3D hydrogel models used in these studies did not control matrix stiffness independently of other confounding parameters that are well known to impact cell function. The resulting lack of precisely engineered hydrogel properties prevents us from disentangling the differential role of various ECM cues, such as stiffness and degradability. Hence, to clarify the reported discrepancies between 2D and 3D findings, hydrogels that offer full and independent control over each of these matrix properties are needed.

While 3D models better recapitulate some of the structural features of natural tissues, the increase in complexity instills a need for more careful material design. In particular, hydrogel swelling should be minimized or eliminated as it imposes a mechanical stimulus upon embedded cells that is distinct from the effect of matrix stiffness.^[^ ^13^ ^]^ This is particularly important since the extent of swelling varies with matrix stiffness in previously employed hydrogel systems,^[^ ^14^ ^]^ thus further complicating the analysis and associated conclusions. Additionally, an ideal hydrogel system should be amenable to local proteolytic cleavage by cells yet hydrolytically stable over long culture periods, so that bulk matrix stiffness remains constant over the course of study.^[^ ^14a^ ^]^ To overcome all of these hurdles, we establish a synthetic hydrogel system in which matrix degradability and stiffness can be tuned independently of one another. A key feature of our approach is that all other key matrix properties including hydrogel swelling and hydrolytic stability remain constant. Exploiting these new possibilities, we elucidate the surprisingly distinct roles of matrix stiffness and degradability in regulating not only cell‐ECM interactions in 3D, but in turn stem cell differentiation outcomes.

## 2 Results and Discussion

**Figure 1:**
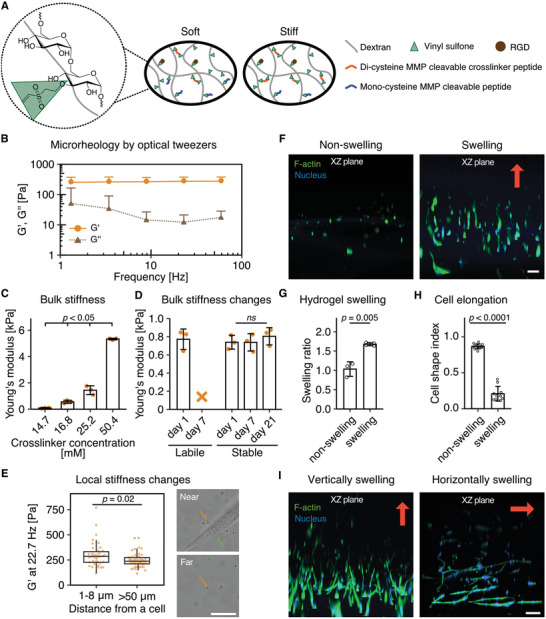
Hydrolytically stable and nonswelling hydrogels are necessary to study cellular stiffness sensing in 3D. A) Scheme of DexVS hydrogel model. DexVS is reacted with the cell‐adhesive peptide cRGD and crosslinked through MMP‐cleavable peptides. B) Optical tweezers measurements of the storage (G') and loss (G'') modulus of hMSC encapsulated DexVS hydrogels crosslinked with 21.0 × 10^−3^ m di‐cysteine‐HD and 29.4 × 10^−3^ m mono‐cysteine‐HD from at least 50 different beads measured on *n* = 3 independent samples. All data are presented as a mean + s.d. C) Young's modulus of DexVS hydrogels as a function of crosslinker concentration, as measured by nanoindentation (*n* = 3 independent samples). D) Young's modulus of hMSC encapsulated hydrolytically labile DexMA hydrogels and stable DexVS with 21.0 × 10^−3^ m peptide crosslinker after 1, 7, and 21 days of culture (*n* = 3 independent samples). The orange X indicates that the hydrogel was fully hydrolyzed after 7 days in cell culture medium. E) Storage modulus of hMSC encapsulated DexVS hydrogels crosslinked with 21.0 × 10^−3^ m di‐cysteine‐HD and 29.4 × 10^−3^ m mono‐cysteine‐HD, measured by optical tweezers (at 22.7 Hz) within 1–8 µm near cells and >50 µm away from cells. The orange arrow indicates beads measured, while the green arrow indicates cells. F) Morphology of hMSCs encapsulated in nonswelling versus swelling soft (≈0.1 kPa) hydrogels (*XZ* plane shown, the red arrow indicates the swelling direction). G) Hydrogel swelling ratio of the nonswelling versus swelling soft (≈0.1 kPa) hydrogels. (*n* ≥ 3 independent samples). H) Cell shape index of hMSCs encapsulated in the nonswelling versus swelling soft (≈0.1 kPa) hydrogels. (*n* ≥ 10 cells). I) Cells elongate along the main axis of swelling (indicated by red arrows). Composite fluorescence images showing F‐actin (green) and nuclei (blue) (scale bar, 100 µm) (*XZ* plane shown). Hydrogel swelling in (F–I) was controlled by the hydrophilicity of the crosslinker peptide. All data are presented as a mean ± s.d. except for (E) as box‐and‐whisker plots (box, 25–75%; bar‐in‐box, median; whiskers, the largest or smallest point comprised within 1.5× of the interquartile range from both edges).

To study ECM mechanosensing in 3D environments, we built upon a previously developed synthetic, linearly elastic hydrogel cell‐encapsulation model based upon vinyl sulfone functionalized dextran (DexVS).^[^ ^15^ ^]^ This hydrogel consists of a protein absorption resistant and cell inert polysaccharide backbone that can be easily functionalized with the cell adhesive peptide cyclic RGD (cRGD) through Michael‐type addition (**Figure** **1**A; Figure S1, Supporting Information).^[^ ^16^ ^]^ Crosslinking DexVS backbones with matrix metalloproteinase (MMP)‐cleavable di‐cysteine peptides generates solid linearly elastic hydrogels (Figure 1B) susceptible to cellular degradation via MMP proteolysis,^[^ ^17^ ^]^ a pre‐requisite for the spreading and migration of cells encapsulated within upon hydrogel crosslinking. Hydrogel stiffness (Figure 1C) was modulated by tuning the ratio of di‐cysteine to mono‐cysteine end‐modified MMP‐cleavable peptides, thereby maintaining the chemical properties (e.g., hydrophilicity, polymer content) of hydrogels despite variations in hydrogel stiffness. Using this approach, we synthesized hydrogels with Young's moduli ranging from 0.1 to 6 kPa, thereby spanning the stiffness range that cells have been previously reported to differentially respond to in 3D hydrogels.^[^ ^18^ ^]^

While hydrogel crosslinks must be locally cleaved by encapsulated cells to generate space for cell spreading, studies of ECM mechanosensing in 3D also require materials whose bulk stiffness remains constant throughout the entire culture period so that the resulting cell response can be directly attributed to a particular factor of interest. Many hydrogel systems developed to date are modified with hydrolytically labile chemical functionalities,^[^ ^4^, ^19^ ^]^ such as methacrylates,^[^ ^20^ ^]^ whose bulk stiffness decreases over the course of several days to weeks due to ester hydrolysis. For example, our previously developed methacrylated dextran (DexMA) hydrogels significantly softened over the course of a few days (Figure 1D). To overcome this problem, we chose vinyl sulfone functionalized dextran as a hydrolytically stable base material and indeed, hydrogel bulk stiffness remained unchanged after three weeks of culture with encapsulated hMSCs (Figure 1D). Whereas cells within hydrolytically stable DexVS hydrogels were able to fully spread over this period, cells encapsulated in hydrolytically labile DexMA hydrogels of the same initial stiffness spread significantly less at this time point, presumably due to diminishing bulk stiffness as a result of hydrolytic degradation (Figure 1D, Figure S2A,B, Supporting Information). While hydrogel bulk stiffness has been established as a major parameter dictating 2D mechanosensing, cells likely probe the mechanical feedback of their local surroundings on a micrometer scale. Whether cell‐mediated hydrogel crosslink cleavage reduces stiffness locally or throughout the bulk is not known; as such, characterizing changes in matrix mechanics spatially with respect to embedded cells is critical to identifying a stiffness response in 3D. In order to mechanically characterize regions of the hydrogel proximal and distal to embedded cells, we performed optical tweezers‐based microrheology. We mapped the hydrogel stiffness local to (within 1–8 µm distance) and further away (> 50 µm distance) from cells, and importantly, did not find a decrease in stiffness in close proximity to the cell (Figure 1E; Figure S2C, Supporting Information). Instead, we even observed a slight increase in stiffness. This indicates that cells only cleave the crosslinks in direct proximity to their membrane, suggesting that only the nanoenvironment of the cell is subject to proteolytically mediated changes in stiffness. Since cells are able to sense up to 10–20 µm into soft hydrogels,^[^ ^21^ ^]^ the mechanical feedback that they experience upon probing the matrix can therefore be considered constant over the culture period in these hydrogels.

Isolating the effects of matrix stiffness on cell function requires the removal of confounding mechanical cues that may covary with stiffness, such as the swelling behavior of commonly used hydrogels composed of hydrophilic polymer networks.^[^ ^14^ ^]^ Previous synthetic hydrogel systems created for 3D cell encapsulation have been purposely designed to undergo pronounced swelling because augmented hydrogel pore size facilitates nutrient transport throughout the polymer network to support cell survival and metabolism. However, we hypothesized that hydrogel swelling following cell encapsulation may generate tensile forces that could themselves influence cell spreading independent of matrix stiffness. To test this hypothesis, cells were encapsulated within soft hydrogels (≈0.1 kPa Young's modulus) whose swelling behavior was defined by polymer backbone hydrophilicity.^[^ ^3a^ ^]^ Specifically, swelling hydrogels were generated either by tuning crosslinker hydrophilicity or by coupling highly hydrophilic thiolated poly(ethylene glycol) sidechains to DexVS through Michael‐type addition during the final crosslinking step. Samples were cast inside cylindrical wells with open tops to restrict post‐crosslinking hydrogel swelling to the vertical axis. When hMSCs were encapsulated in soft, nonswelling hydrogels, cells adopted a round morphology similar to the phenotype observed on soft 2D substrates,^[^ ^2b^ ^]^ whereas hMSCs or human dermal fibroblasts (HDFs) encapsulated in soft, swelling hydrogels (Figure 1G; Figure S3A,B, Supporting Information) displayed highly elongated morphologies (Figure 1F–H; Figure S2D and S3C,D). Importantly, when the axis of swelling was changed by altering the locations of rigid, confining boundaries, hMSCs consistently spread along the axis of swelling (Figure 1I; Figure S3C, Supporting Information). This clearly demonstrates that mechanical forces arising from hydrogel swelling influence cell spreading, similar to what has been observed for cells stretched on flexible substrates.^[^ ^22^ ^]^

**Figure 2:**
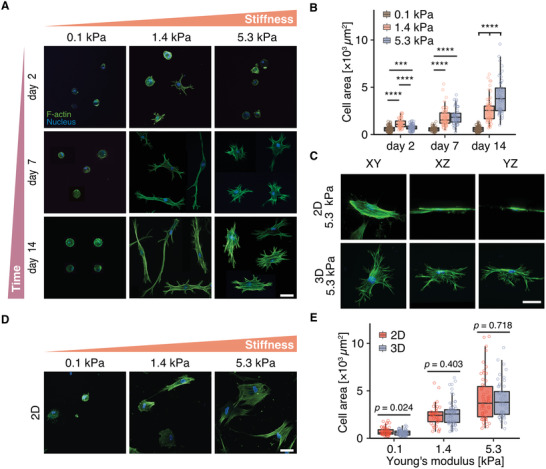
hMSC spreading increases with hydrogel stiffness at equilibrium spread state. A) Morphology of hMSCs cultured within DexVS hydrogels crosslinked with 10.1 × 10^−3^, 25.2 × 10^−3^, and 50.4 × 10^−3^ m MMP‐cleavable peptides for 2, 7, and 14 days. B) Quantification of cell spread area for conditions shown in (A). C) 3D orientation of hMSCs cultured within 5.3 kPa DexVS hydrogels. D) hMSCs cultured on 2D DexVS hydrogels of different stiffness for 24 h (where maximum spreading was reached). E) Quantification of cell spread area for conditions shown in (A) and (D), respresenting the stage at which cells reached maximum spreading in 3D (A, after 14 days of culture) and 2D (D, after 24 h of culture), respectively. Overall statistical analysis was performed with one‐way ANOVA test (*p* < 0.001). Composite fluorescence images showing F‐actin (green) and nuclei (blue). All data are presented as box‐and‐whisker plots (box, 25–75%; bar‐in‐box, median; whiskers, the largest or smallest point comprised within 1.5× of the interquartile range from both edges). Two‐tailed unpaired Student's *t*‐test without adjustment was performed for individual comparisons. *** *p* < 0.001, **** *p* < 0.0001. Exact *p* values in (C) are as follows: Day 2: 0.1 kPa versus 1.4 kPa *p* = 2.78 × 10^−18^, 0.1 kPa versus 5.3 kPa *p* = 2.21 × 10^−4^, 1.4 kPa versus 5.3 kPa *p* = 1.56 × 10^−9^; Day 7: 0.1 kPa versus 1.4 kPa *p* = 8.12 × 10^−14^, 0.1 kPa versus 5.3 kPa *p* = 1.31 × 10^−16^, 1.4 kPa versus 5.3 kPa *p* = 0.454; Day 14: 0.1 kPa versus 1.4 kPa *p* = 1.68 × 10^−28^, 0.1 kPa versus 5.3 kPa *p* = 3.04 × 10^−34^, 1.4 kPa versus 5.3 kPa *p* = 1.55 × 10^−6^, *n* ≥ 50. Scale bar, 50 µm.

We next used our nonswelling hydrogel system (Figure S4, Supporting Information) to examine how hMSCs respond to changes in 3D matrix stiffness. After 2 days in culture, we observed a bimodal response of projected cell spread area to matrix stiffness, where cells spread maximally at an intermediate hydrogel stiffness (**Figure** **2**A,B). This trend held consistent across multiple mesenchymal cell types, as confirmed with HDFs (Figure S5A,B, Supporting Information). The initial difference in spread area comparing ≈0.1 and 1.4 kPa hydrogels can be explained by the well‐established stiffness effects described on 2D hydrogel surfaces, where cells experience increased mechanical resistance from stiffer substrates leading to focal adhesion formation, actomyosin activity, and cell elongation.^[^ ^2^, ^5^, ^8^ ^]^ However, at a higher stiffness of 5.3 kPa, cell spreading appeared to be impaired. Importantly, we noticed that across the entire stiffness range, cell spreading was overall rather limited when compared to cells seeded atop identical hydrogels with the same composition and stiffnesses (Figure 2D). We therefore speculated that longer culture times would be required to reach equilibrium spreading in 3D, due to the requirement for matrix degradation and the associated generation of open space required for cell spreading in 3D. In fact, our recent studies demonstrate that in 3D hydrogel environments, changes in matrix crosslinking not only alter matrix stiffness, but also influence how rapidly cells can degrade the surrounding hydrogel in order to spread and migrate during angiogenic sprouting.^[^ ^3a^ ^]^ Hydrogel degradability, or the rate at which cells can solubilize a unit volume of surrounding hydrogel, is lower in highly crosslinked, stiffer matrices, compared to lightly crosslinked, softer matrices. Indeed, when we increased culture time to allow cells to achieve an equilibrium spreading state, we observed a monotonic increase in cell spreading with increasing matrix stiffness. Interestingly, despite obvious differences in cell morphology between 2D and 3D culture (Figure 2C), the projected spread area at equilibrium in 3D was comparable to that at the same hydrogel stiffness in 2D (where cells maximally spread within one day) (Figure 2A,D,E).^[^ ^5b^ ^]^ This suggests that the final spread state of the cell is determined by the stiffness of the matrix independent of culture dimensionality, but concurrent changes in matrix degradability determine spreading kinetics.

**Figure 3:**
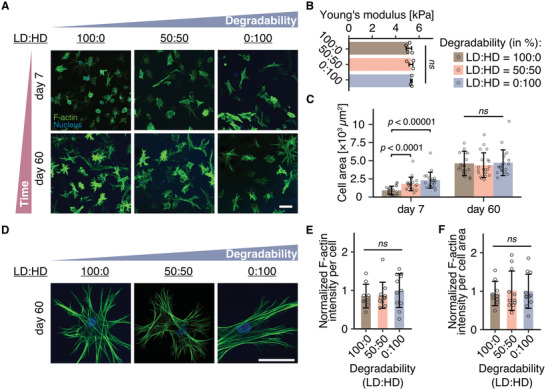
Matrix degradability regulates cell spreading kinetics, while matrix stiffness determines the final spread state of hMSCs. A) Morphology of hMSCs cultured within hydrogels of varying degradability for 7 and 60 days. Degradability was tuned by changing the susceptibility of the peptide crosslinker toward cell released MMPs. LD: low degradability peptide crosslinker. HD: high degradability peptide crosslinker. B) Young's modulus of DexVS hydrogels crosslinked with mixtures of di‐cysteine‐LD and di‐cysteine‐HD peptides after equilibration in cell culture medium for 24 h, as measured by nanoindentation (*n* ≥ 3 independent samples). All data are presented as a mean ± s.d. Two‐tailed unpaired Student's t‐test without adjustment was performed for individual comparisons. ns, not significantly different (p > 0.05). C), Quantification of cell spread area for conditions shown in (a) (*n* = 20 cells were analyzed each). Overall statistical analysis was performed with one‐way ANOVA test (*p* < 0.001). D) Representative high magnification confocal images of hMSCs cultured in hydrogels with varying degradability at day 60. Composite fluorescence images showing F‐actin (green) and nuclei (blue). E,F) Normalized intensity of F‐actin per cell (E) and per cell area (F) at day 60. *n* ≥ 10 cells. All data are presented as a mean ± s.d. Two‐tailed unpaired Student's *t*‐test without adjustment was performed for individual comparisons. ns not statistically different. Scale bar, 100 µm

Matrix degradability is not only regulated by crosslink density or matrix stiffness, but also by the susceptibility of the particular crosslink sequence to cleavage by cell‐produced enzymes.^[^ ^17a^ ^]^ In order to study the impact of matrix degradability on cell spreading without altering matrix stiffness or degree of crosslinking, we generated identical hydrogels replacing the MMP‐cleavable crosslinker sequence with one of diminished MMP‐susceptibility;^[^ ^17a^ ^]^ the nonswelling behavior as well as crosslink density and stiffness of these hydrogels was kept consistent (**Figure** **3**; Figure S6, Supporting Information ). After 7 days in culture, we observed decreased cell spreading with decreased hydrogel degradability; however, at later time points when cells were allowed to achieve equilibrium spreading, cell spread area proved to be independent of matrix degradability (Figure 3A,C). Similarly, stress fiber formation in cells at late culture points did not differ between hydrogels of varying degradability (Figure 3D–F). Together, these results clearly demonstrate that in 3D hydrogels, the kinetics of cell spreading are regulated by matrix degradability, whereas matrix stiffness critically defines the eventual final spread state of the cell.

**Figure 4:**
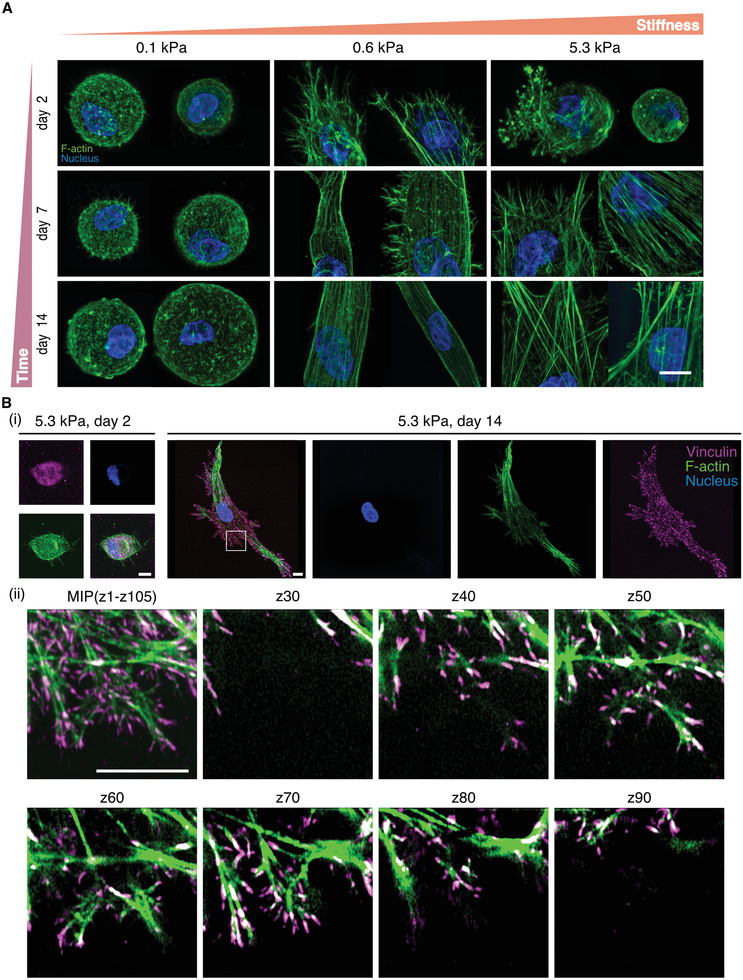
Actin stress fiber and focal adhesion formation correlate with the spread state of the cell. A) Stress fiber formation in hMSCs cultured within DexVS hydrogels crosslinked with 10.1 × 10^−3^, 16.8 × 10^−3^, and 50.4 × 10^−3^ m MMP‐cleavable peptides for 2, 7, and 14 days. B) Focal adhesion formation, as visualized by antibody staining against vinculin, in hMSCs cultured in 5.3 kPa hydrogels for 2 and 14 days. Images in i) are 3D maximum intensity projections (MIP) of entire cells, whereas ii) shows different z planes of the framed area in i) (images are separated by a step size of 1.92 µm), demonstrating that focal adhesions are fully distributed across all three dimensions. Composite fluorescence images showing F‐actin (green), nuclei (blue) and vinculin (magenta). Scale bar, 10 µm.

We next investigated how changes in 3D matrix stiffness influence the formation of stress fibers, well‐established to be critical transducers of mechanical ECM cues to cell signaling events and transcriptional activity.^[^ ^23^ ^]^ We found that at early time points, spread cells cultured in hydrogels of intermediate stiffness possessed stress fibers, whereas round cells cultured in soft and stiff gels only formed punctate F‐actin clusters (**Figure** **4**A; Figure S7A,B, Supporting Information). This indicates that stress fiber formation requires cell spreading, and if spreading is inhibited either due to low matrix stiffness or low matrix degradability, stress fibers cannot form. To further confirm this observation, we again allowed cells to reach equilibrium spreading by extending the culture time, and observed increased stress fiber formation commensurate with increased cell spreading in stiffer matrices, in line with what has previously been described for cells cultured on 2D substrates (Figure 2D and Figure 4A).^[^ ^5^ ^]^ Moreover, the degree of stress fiber formation consistently correlated with the extent of focal adhesion formation, as shown by the clustering of vinculin (Figure 4Bi,ii; Figure S7C, Supporting Information). Vinculin localization to focal adhesions was fully distributed across all three dimensions (Figure 4Bii) and most prominent in well‐spread cells within stiff hydrogels. Together, these experiments uncover matrix stiffness as an important regulator of stress fiber formation in 3D, however, hydrogel platforms that concurrently modulate matrix stiffness and degradability may obfuscate such stiffness responses by hampering cell spreading required for focal adhesion and stress fiber formation.

**Figure 5:**
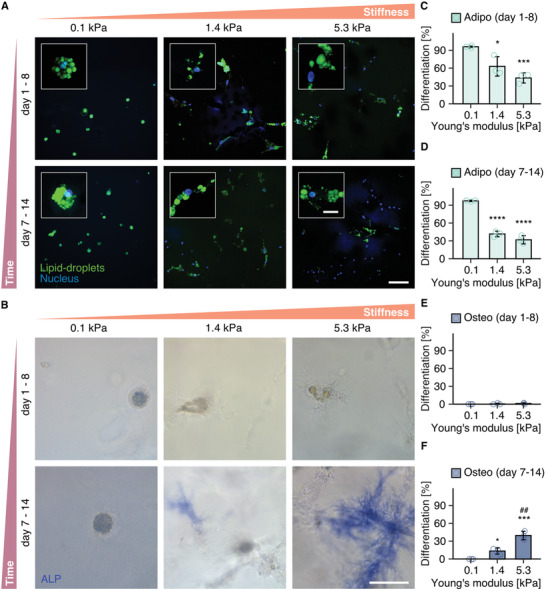
hMSC differentiation toward osteoblasts and adipocytes is cell spreading and matrix stiffness dependent. A,B), Differentiation of hMSCs toward A) adipocytes, visualized by lipid droplet staining with Bodipy (green), nuclei (blue) and B) osteoblasts, visualized by staining of ALP activity within hydrogels crosslinked with 12.6 × 10^−3^, 25.2 × 10^−3^, and 50.4 × 10^−3^ m MMP‐cleavable peptides. Cells were differentiated in a 1:1 mixed adipo/osteo induction medium either from day 1 to 8, or from day 7 to 14. (C, D, E, F), Percentage of hMSCs differentiating toward adipogenic (C, D) or osteogenic (E, F) lineage (*n* = 50 cells were counted from *n* = 3 independent experiments). All data are presented as a mean ± s.d. Overall statistical analysis was performed with one‐way ANOVA test (*p* < 0.001). Two‐tailed unpaired Student's *t*‐test without adjustment was performed for individual comparisons. Exact *p* values are as follows: C): * *p* = 0.0257, *** *p* = 4.82 × 10^−4^. D): (from left to right) **** *p* = 3.99 × 10^−5^,  **** *p* = 8.54 × 10^−5^, F):  = 0.0101, *p* *## *p* = 6.85 × 10^−3^, *** *p* = 6.65 × 10^−4^. *: as compared to 0.1 kPa hydrogel group. #: as compared to 1.4 kPa hydrogel group (scale bar: 100 µm, scale bar in insets: 20 µm).

To determine if the interplay between matrix stiffness and degradability has consequences for cell functions dependent on cell spreading and adhesion, we examined the differentiation of hMSCs toward adipocyte and osteoblast lineages (**Figure** **5**A–F). Previous work using 2D substrates has established stem cell fate decision‐making to be highly dependent on stiffness where more rigid substrates promote osteogenic differentiation in contrast to softer substrates that encourage adipogenesis.^[^ ^2^, ^5^ ^]^ When hMSCs were induced to differentiate by the addition of a mixed adipo/osteo induction media immediately following 3D encapsulation, only adipocytes identified by Bodipy‐positive lipid droplets were observed 7 days later across all matrix stiffnesses examined. However, when cells were first allowed to spread in growth media for 7 days prior to the introduction of induction media, cells differentiated toward adipocytes in soft environments, but the percentage of hMSCs differentiating toward alkaline phosphatase (ALP) positive osteoblasts increased with increasing matrix stiffness. These results support the model that degradability and matrix stiffness cooperate to determine cell shape, which in turn regulates hMSC differentiation.

## 3 Conclusion

Here, a nonswelling and hydrolytically stable synthetic hydrogel platform enabled us to uncover the distinct roles of matrix degradability and stiffness in 3D, two important microenvironmental cues that are intrinsically coupled in natural ECMs. While matrix degradability modulates the kinetics of cell spreading, matrix stiffness defines cell spreading at equilibrium. In direct contrast to the observations made on 2D hydrogels,^[^ ^5a^ ^]^ recent literature reports have shown that 3D cell spreading decreases with increased matrix stiffness due to changes in matrix degradability.^[^ ^11b^ ^]^ As a result, matrix degradability has been highlighted as the dominant parameter governing cell shape and function in 3D hydrogels.^[^ ^12^ ^]^ This has led to the overall notion that observations from 2D culture experiments may not be transferable to 3D hydrogel settings or native 3D tissues. However, our work highlights that in 3D, matrix stiffness indeed regulates cell shape and differentiation in similar fashion to 2D surfaces, with the caveat that concurrent changes in degradability can obscure such stiffness effects by regulating spreading kinetics. Previous reports only examined cells at time points prior to equilibrium spreading, whereby the influence of matrix stiffness was not observed. Notably, many of the previously explored hydrogel systems were predicated on hydrolytically unstable crosslinks (such as methacrylates),^[^ ^20^ ^]^ which cause hydrogels to degrade and soften with culture due to hydrolysis; as such, cells in these studies never achieved maximal spread states that reflect the initially defined stiffness of the hydrogel. In contrast, hydrolytically stable hydrogels allow cells sufficient time to cleave their local 3D environment and achieve a final spread state similar to what has been noted on 2D hydrogels. Importantly, throughout culture, the local stiffness in direct proximity to the cells does not decrease as a function of MMP secretion, as demonstrated by our optical tweezers‐based microrheology measurements. In turn, we even observe a slight increase in stiffness of about 20% in close proximity of cells, which is accompanied by an increase in the variance of these measurements. This slight increase could be attributed to several factors. For example, the mechanical binding of the stiff cell cortex may increase the apparent measured stiffness with optical tweezers. However, as the cortical stiffness is only slightly above the hydrogel stiffness,^[^ ^5^, ^24^ ^]^ only a marginal effect of adhesion is expected, as confirmed by the measurements. Additionally, optical inhomogeneities introduced by the cell body may lead to an increase in noise that contributes to the observed larger data spread. Besides these systematic errors in the measurement, a real stiffness increase can be explained by recent reports which have shown that cells embedded in 3D hydrogels secrete a layer of ECM proteins.^[^ ^25^ ^]^ Finally, we would like to note that a slight change in stiffness of 20% is likely to have only limited effects on cells, as it is small relative to the stiffness changes of 2–3 fold that have been reported to illicit different cell responses previously.^[^ ^11b^ ^]^ Hence our experiments allow for a full decoupling of hydrogel stiffness and degradability for the analysis of cell spreading and more generally, function.

Furthermore, in 3D, prior studies have reported cells to be maximally spread at low stiffnesses,^[^ ^11b^ ^]^ whereas cells cultured on substrates of this stiffness in 2D remained unspread due to the lack of mechanical support from the substrate.^[^ ^2b^ ^]^ We suggest that the observed spreading at low stiffnesses could be explained by the high degree of swelling that is typically accentuated in soft matrices with low crosslinking, potentially explaining the discrepancy between 2D and 3D settings. Hence, our studies reconcile findings from both in vivo,^[^ ^3^, ^26^ ^]^ as well as 2D substrates with the more recent data from 3D synthetic hydrogel models, and therefore constitute an important step toward a better mechanistic understanding of the interplay between various matrix properties in more complex tissue‐like matrices. While linearly elastic hydrogels, whose stiffness is not affected by the magnitude or rate of cellular deformation, are ideal to understand basic cellular mechanosensing, they do not capture the nonlinear behavior of natural tissues. In particular, viscoelasticity has been demonstrated to be an important parameter regulating cellular mechanosensing.^[^ ^11c^ ^]^ Using hydrogel models based on weak ionic crosslinks that exhibit stress relaxation upon application of cellular strain, it was found that MSC spreading and differentiation is greatly enhanced when cultured in environments with great stress relaxation, mainly due to the mechanical clustering of adhesion ligands. Therefore, a full mechanistic understanding requires a combination of different sets of materials and approaches which complement each other.

Using a modular hydrogel system, in which matrix stiffness and degradability could be tuned independently and in the absence of other covarying and confounding parameters, we were able to uncover the important role of cell shape in regulating stress fiber formation and stem cell differentiation in 3D hydrogels. While matrix degradability and stiffness jointly regulate the extent of cell spreading, the resulting shape of the cells ultimately determines downstream signaling. In this context, it does not seem to make a difference if cells take up a certain shape due to changes in matrix stiffness or degradability; instead, cells integrate multiple ECM signals to define their resulting shape which appears to be predictive of functional behaviors such as stem cell differentiation. This observation supports previous studies elucidating the importance of cell shape changes for cell fate decisions.^[^ ^2^, ^24^, ^27^ ^]^ Together, our studies stress the importance of understanding how microenvironmental cues in 3D environments individually as well as synergistically drive (stem) cell function in order to inform the design of materials for tissue engineering applications.

## 4 Experimental Section

### Reagents

All reagents were purchased from Sigma Aldrich, unless otherwise indicated.

### Adhesive Peptides and MMP‐Cleavable Peptides

The cell adhesive peptide cyclo(RGDfK(C)) (cRGD) was purchased from Peptides International. The matrix metalloproteinase‐cleavable peptide sequences KCVPMSMRGGCK (di‐cysteine‐HD), KCVPMSMRGGGK (mono‐cysteine‐HD), KCGPQGIAGQCK (di‐cysteine‐LD), and KCGPQGIAGQGK (mono‐cysteine‐LD) were custom synthesized by GenScript and provided as hydrochloride salt (purity > 95%).

### Antibodies

The mouse monoclonal anti‐vinculin antibody was purchased from Sigma Aldrich (#V9131). Alexa Fluor 555 conjugated donkey anti‐mouse secondary antibody was obtained from Life Technology (#A31570).

### Synthesis of Methacrylated Dextran (DexMA)

DexMA was prepared according to a previously published procedure.^[^ ^3^, ^20^ ^]^ In brief, dextran (20 g, MP Biomedicals, MW 86 000 Da) and 4‐dimethylaminopyridine (2 g) were dissolved in anhydrous dimethyl sulfoxide (100 mL). Glycidyl methacrylate (24.6 mL) was added under stirring, the mixture was heated to 45°C and the reaction allowed to proceed for 24 h. Next, the solution was precipitated into cold 2‐propanol (1 L, VWR). The crude product was collected, re‐solubilized in Milli‐Q water and dialyzed against Milli‐Q water for three days. A methacrylate/dextran repeat unit ratio of 0.7 was determined by ^1^H NMR spectroscopy.

### Synthesis of Dextran Vinyl Sulfone (DexVS)

DexVS was synthesized as previously reported.^[^ ^15^ ^]^ In brief, divinyl sulfone (2.48 mL, purity > 97%) was added dropwise to a solution of dextran (2.0 g, MP Biomedicals, MW 86 000 Da) in aqueous sodium hydroxide (0.1 M, 100 mL) under vigorous stirring at room temperature. After 5 min, the reaction was stopped by adjusting the pH to 5 through the addition of hydrochloric acid solution (2.4 M). The mixture was dialyzed (SnakeSkin™ Dialysis Tubing, Life Technologies, 10 kDa) against Milli‐Q ultrapure water at room temperature, and the water was exchanged twice a day for three days. The final product was obtained through lyophilization. A vinyl sulfone/dextran repeat unit ratio of 0.5 was determined by ^1^H NMR spectroscopy.

### Cell Culture

Human bone marrow derived mesenchymal stromal cells (hMSCs) were obtained from ATCC and PromoCell. Cells were maintained and expanded in growth medium, containing low glucose DMEM (Thermo Fisher Scientific) supplemented with 10% FBS, 1% L‐glutamine, and a 1% penicillin‐streptomycin solution. Passages 4 and 8 were used for differentiation experiments and analysis of single cells, respectively. Human dermal fibroblasts (HDFs) were purchased from ATCC. HDFs were maintained and expanded in cell culture medium, containing high glucose DMEM (Corning) supplemented with 10% FBS, 1% L‐glutamine, and a 1% penicillin‐streptomycin solution. Passage 7 cells were used for all experiments.

### Cell Encapsulation within DexVS and DexMA Hydrogels

All reagents were dissolved in PBS. All solutions were cooled with ice (‐20°C) prior to and during reaction.

A neutralized solution of cRGD (final concentration of 1.5 × 10^−3^ m) was reacted with DexVS (final concentration of 4.4% w/v) or DexMA (final concentration of 4.4% w/v) via Michael‐type addition at pH ≈ 7.5. A mixture of variable concentrations of di‐cysteine peptide crosslinker (di‐cysteine‐HD or di‐cysteine‐LD; final concentrations of 10.1 × 10^−3^, 25.2 × 10^−3^, and 50.4 × 10^−3^ m) and mono‐cysteine peptide (mono‐cysteine‐HD or mono‐cysteine‐LD; final concentrations of 40.3 × 10^−3^, 25.2 × 10^−3^, and 0 × 10^−3^ m, respectively), in which the total concentration of MMP‐cleavable peptide was kept constant at 50.4 × 10^−3^ m, was added. The ice‐cold precursor solution was neutralized with an aqueous solution of NaOH (0.25 M) to pH ≈ 7.5 to initiate hydrogel gelation. Immediately, hMSCs resuspended in pure FBS were added at a final density of 1 × 10^5^ cells mL^−1^. Finally, the forming hydrogels were incubated for an additional 30 min at room temperature to allow for full gelation. The hydrogel cell cultures were maintained in growth medium in a cell culture incubator with constant humidity at 37°C and 5% CO_2_. Medium was exchanged every three days. Samples were fixed after 2, 7 or 14 days of culture.

### Mechanical Characterization by Nanoindentation

Young's moduli of the hydrogels were characterized using a nanoindenter (Piuma, Optics 11, Netherlands) at day 1, 7 and 21. A cantilever with a spring constant of 0.03 N m^−1^ and the bead diameter of 60 um was used. The Young's modulus of each hydrogel was averaged from at least 10 indentations on 3 independent hydrogels of 6 mm diameter immersed in PBS supplemented with 2% FBS. Indentation curves were fitted with a Hertz contact model.

### Optical Tweezers Measurements

Hydrogels composed of di‐cysteine‐HD (21.0 × 10^−3^ m), mono‐cysteine‐HD (29.4 × 10^−3^ m) and cRGD (1.5 × 10^−3^ m) with embedded hMSCs (density of 2 × 10^5^ cells ml^−1^) and 1 µm polystyrene beads (1:100 diluted) were cultured for 7 days prior to measurements. At least 50 measurements near (1–8 µm away) and far (>50 µm away) from cells were taken from 3 independent hydrogel samples of 400 µm thickness in cell culture medium. Only beads at least 100 µm above the coverslip were measured. The tweezers setup was described previously.^[^ ^28^ ^]^ Briefly, a polystyrene particle was trapped in the focus of an infrared laser (*λ* = 1064 nm; IPG Photonics) while the laser position was controlled by a pair of acousto‐optic XY‐deflectors (DTSX‐400‐1064; AA Opto‐Electronic). The laser light was coupled into an inverted microscope (Eclipse Ti‐e; Nikon) and focused in the object plane by a water immersion objective (60x, NA = 1.2; Nikon). A condenser positioned above the object was used to collect the infrared light. A force sensor (Lunam T‐40i; Impetux Optics) was used to measure the applied force. For the detection of the particle displacement, a second infrared laser (*λ* = 976 nm, Thorlabs) was used. The back focal plane of the condenser was imaged on a position‐sensitive diode (Thorlabs). The detection laser signal was calibrated by scanning the stage that holds the object through the laser beam via a piezo element (PXY 80 D12, piezosystem Jena). A characteristic slope was then fit to the scan, which was subsequently used to convert the displacement signal from volt into micrometres. An individual scan was used for each measurement. All hardware was controlled using a home‐written LabVIEW program. During a measurement, the 1064‐laser was oscillated with an amplitude of 0.5 µm and a frequency between 0.2 and 5000 Hz. A Fourier transformation was then taken of the measured force and displacement to select the force $\tilde{F}\left(f\right)$ and the displacement $\tilde{x}\left(f\right)$ at the applied frequency. From their ratio the response function $\tilde{\chi }\left(f\right)$ was calculated:

(1) $$\tilde{\chi }\;\left(f\right)=\frac{\tilde{x}\left(f\right)}{\tilde{F}\left(f\right)}$$

The response function was then used to calculate the complex shear modulus G*(*f*) = *G*′ (*f*) + *i* *G*′′(*f*):

(2) $$G^{*}\left(f\right)=\frac{1}{6\pi r\tilde{\chi }\left(f\right)}$$

where *r* was the radius of the polystyrene particle.

### Hydrogel Swelling

Hydrogel swelling was controlled by tuning the hydrophilicity of the dextran backbone through two independent approaches. Our first method relied on the modulation of hydrophilicity of the MMP‐cleavable crosslinker peptide. For this purpose, we first characterized literature‐reported, cell‐cleavable peptide sequences with regards to their hydrophilicity. Specifically, we calculated the grand average of hydropathy (GRAVY), a computational value that has previously been established as a measure of hydrophilicity of proteins and peptides based on their amino acid sequence.^[^ ^29^ ^]^ Using the openly available Expasy tool (Swiss Institute of Bioinformatics), we found that the MMP‐cleavable peptide KCVPMSMRGGCK (HD, GRAVY: ‐0.208) is less hydrophilic than the crosslinker peptide KCGPQGIAGQCK (LD, GRAVY: ‐0.525). We have therefore chosen these two sequences to control the swelling behavior of soft DexVS hydrogels, which were prepared as follows: All hydrogels contained 4.4% w/v DexVS and 1.5 × 10^−3^ m cRGD. The nonswelling hydrogel was crosslinked by a mixture of 10.1 × 10^−3^ m di‐cysteine HD and 40.3 × 10^−3^ m mono‐cysteine HD, and the swelling hydrogel contained 10.1 × 10^−3^ m di‐cysteine LD and 40.3 × 10^−3^ m mono‐cysteine LD. During gelation, 2 and 5 × 10^5^ hMSCs (or HDFs) mL^−1^ were encapsulated in the nonswelling and swelling hydrogels, samples were cultured for 3 days to reach equilibrium swelling, followed by analysis of cell elongation along the axis of swelling. To restrict swelling to the vertical axis, hydrogels were placed in cylinder‐shaped PDMS (Dow Corning, 10:1 base: curing agent) wells. To achieve horizontal swelling, hydrogels were attached to an underlying glass coverslip only. The swelling ratio was calculated by dividing the hydrogel wet mass after swelling by the wet mass before swelling. For this purpose, empty PDMS wells (5 mm diameter and 1.5 mm thickness) were weighed before and after addition of the pre‐gel solution to obtain the initial mass of the hydrogel. After equilibration in medium, the samples were taken out of the buffer, excess solution on the hydrogel surface was carefully removed with a tissue, and the hydrogel‐laden wells were weighed again to determine the weight after swelling. All experiments were repeated three times.

In an alternative approach, we tuned hydrogel hydrophilicity by coupling hydrophilic poly(ethylene glycol) (PEG) sidechains to the dextran backbone. All hydrogels contained 4.4% w/v DexVS and 1.5 × 10^−3^ m cRGD. The nonswelling hydrogel was crosslinked by a mixture of 14.7 × 10^−3^ m di‐cysteine HD and 35.7 × 10^−3^ m mono‐cysteine HD, and for the swelling hydrogel, 35.7 × 10^−3^ m O‐(2‐Mercaptoethyl)‐O’‐methylpolyethylene glycol (MW 2000 Da) was added to the crosslinker mixture containing 14.7 × 10^−3^ m di‐cysteine HD. During gelation, hMSCs were encapsulated in the nonswelling and swelling hydrogels at a density of 2 × 10^5^ hMSCs mL^−1^, and samples were processed and analyzed as described above.

### Hydrogel Degradability

The hydrogel degradability toward cellular MMPs was tuned through the amino acid sequence of MMP‐cleavable crosslinker peptides. 5 kPa hydrogels (DexVS, final concentration of 4.4% w/v) were crosslinked with mixtures of low degradability (di‐cysteine‐LD) and high degradability (di‐cysteine‐HD) peptides (ratios of 100:0, 50:50, 0:100; total peptide concentration of 50.4 × 10^−3^ m). During gelation, hMSCs (1 × 10^5^ cells mL^−1^) were encapsulated in the hydrogels. Samples were cultured for 7 and 60 days, followed by analysis of cell spread area.

### Fluorescent Staining, Microscopy, and Image Analysis

To visualize the F‐actin cytoskeleton, cells embedded in hydrogels were fixed with 4% paraformaldehyde (Thermo Fisher Scientific) at room temperature for 30 min. Cell nuclei and the F‐actin cytoskeleton were stained with Hoechst 33 342 (Thermo Fisher Scientific, 1:1000) and Alexa Fluor 488 phalloidin (Thermo Fisher Scientific, 1:1000) in PBS at room temperature overnight. Samples were inverted and imaged by Andor Dragonfly high speed spinning disk confocal microscopy at 10×, 40× and 60× magnifications. Images are presented as maximum intensity projections. For quantification of cell spread area, laser exposure time and gain were kept constant for all samples in one experiment. Quantification was performed by ImageJ using 10× magnification images to avoid biased selection of cells at higher magnifications.

To visualize focal adhesions, samples were fixed with 4% paraformaldehyde at room temperature for 30 min, cut in half and incubated in 0.5% Triton X‐100 (Thermo Fisher Scientific) overnight at room temperature. Then, samples were blocked with 10% FBS in PBS for 1 h, and incubated with mouse monoclonal anti‐vinculin primary antibody (1:50 in blocking buffer) for 1 h at room temperature. Finally, samples were washed with 0.1% Tween 20 in PBS three times, incubated with a solution containing Alexa Fluor 555 conjugated donkey anti‐mouse secondary antibody (Life Technology) (1:1000), Hoechst 33 342 (1:500) and Alexa Fluor 488 phalloidin (1:500) for 1 h at room temperature. Samples were washed with 0.1% Tween 20 in PBS three times prior to imaging. Actin stress fibers and vinculin were imaged from the cross‐section side with Airyscan super‐resolution microscopy (Zeiss 880 laser scanning confocal microscope) using a 40× objective. The F‐actin intensity per cell was measured by ImageJ with 40× images of maximum intensity projection. The F‐actin intensity per cell was the F‐actin intensity per cell divided by the respective cell area.

### Differentiation of hMSCs

Hydrogels containing 1 × 10^5^ hMSCs mL^−1^ were first incubated in growth medium for 1 or 7 days, followed by a 7‐day differentiation period in osteogenic and adipogenic induction medium mixed at 1:1 ratio. The osteogenic medium was prepared from growth medium supplemented with b‐glycerophosphate (10 × 10^−3^ m), L‐ascorbic acid (250 µM), and dexamethasone (0.1 µM), while the adipogenic medium was supplemented with 3‐isobutyl‐1‐methylxanthine (500 µM), insulin (10 µg ml^−1^), indomethacin (200 µM) and dexamethasone (1 µM). Differentiation medium was exchanged every three days. At the end of the experiment, samples were fixed with 4% paraformaldehyde for 30 min at room temperature. To assess osteogenic lineage specification, encapsulated hMSCs were stained for ALP activity using the Leukocyte Alkaline Phosphatase Kit (Simga, #86C). To ensure sufficient diffusion of the staining reagents, the staining process was performed three times. For adipogenesis, lipid droplets were stained with Bodipy^TM^ 493/530 (Invitrogen, 1:1000), nuclei were counterstained with Hoechst 33 342 (1:1000) in PBS at room temperature overnight. The osteogenic marker ALP was imaged and visualized by an inverted brightfield microscope (Leica DMi1) with a camera (Leica MC120 HD) at 40× magnification, while lipid droplets were imaged by confocal microscopy at 10× and 40× magnifications (Dragonfly, Andor). Percentages of ALP positive and lipid droplet containing cells were quantified relative to the total number of cells per image.

### Statistical Analysis

No statistical method was used to predetermine sample size. No outlier was excluded. Statistical significance and *p* values were determined using one‐way ANOVA test via R studio and two‐tailed unpaired Student's *t*‐tests without any adjustments via Microsoft Excel. The *p* values and sample size of each experiment were indicated in the related figure legend. *p* < 0.05 was considered as statistically significant.

## Conflict of Interest

The authors declare no conflict of interest.

## Supporting information

Supporting Information

## Data Availability

The data that support the findings of this study are available from the corresponding author upon reasonable request.
