# Supplementary material for: Nonswelling and Hydrolytically Stable Hydrogels Uncover Cellular Mechanosensing in 3D
Source: Adv Sci (Weinh). 2022 Feb 20;9(12):2105325. doi: 10.1002/advs.202105325 (PMC9036035; doi:10.1002/advs.202105325)
Supplement: Supplementary file 1 — Supporting Information [file ADVS-9-2105325-s001.pdf]

ADVANCED SCIENCE
Open Access

Supporting Information

for *Adv. Sci.*, DOI 10.1002/advs.202105325

Nonswelling and Hydrolytically Stable Hydrogels Uncover Cellular Mechanosensing in 3D

*Hongyan Long, Bart E. Vos, Timo Betz, Brendon M. Baker and Britta Trappmann**

Supporting Information

Nonswelling and hydrolytically stable synthetic hydrogels uncover cellular mechanosensing in 3D

*Hongyan Long, Bart E. Vos, Timo Betz, Brendon M. Baker, Britta Trappmann**

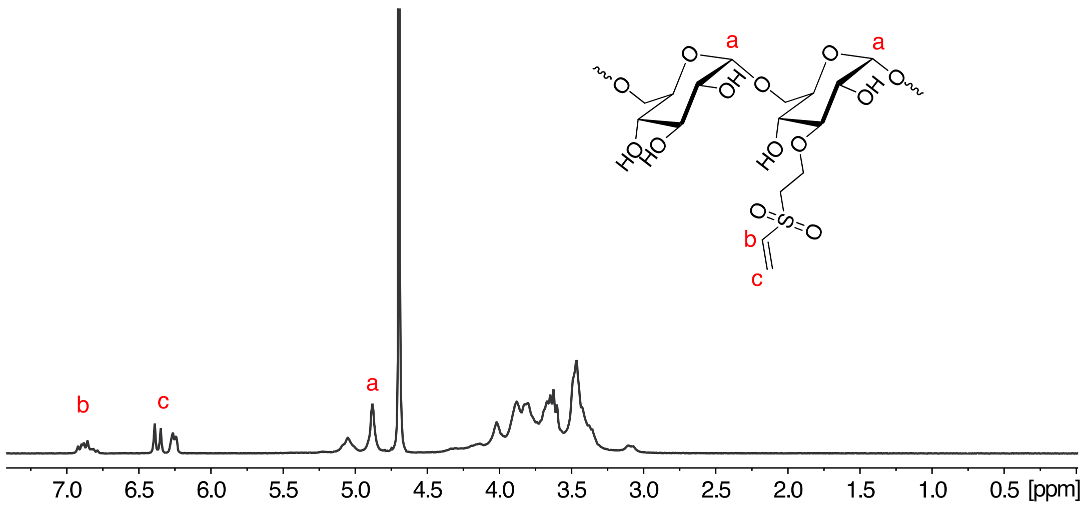

Figure S1. ^{1}H-NMR spectrum of DexVS.

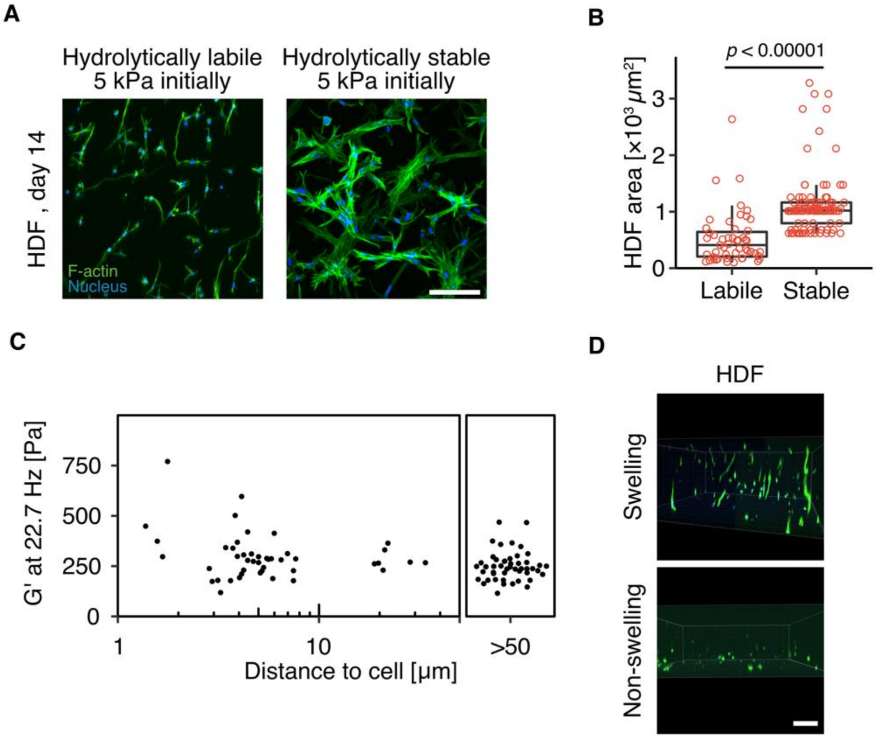

Figure S2. (A), Morphology of human dermal fibroblasts (HDFs) cultured for 14 days in hydrolytically labile (DexMA) and hydrolytically stable (DexVS) hydrogels crosslinked with 50.4 mM MMP-cleavable peptides. (B), Cell spread area of HDFs in (A). Data are presented as box-and-whisker plots (box, 25–75%; bar-in-box, median; whiskers, the largest or smallest point comprised within 1.5× of the interquartile range from both edges). Two-tailed unpaired Student's *t*-test without adjustment was performed for individual comparisons. $n > 50$. (C), Storage modulus of hMSC encapsulated DexVS hydrogels crosslinked with 21.0 mM MMP-cleavable peptides, measured by optical tweezers-based microrheology. (D), Morphology of HDFs in swelling and non-swelling DexVS hydrogels (~ 0.1 kPa) at day 3. Composite fluorescence images showing F-actin (*green*) and nuclei (*blue*). *Scale bars*, 200 μm.

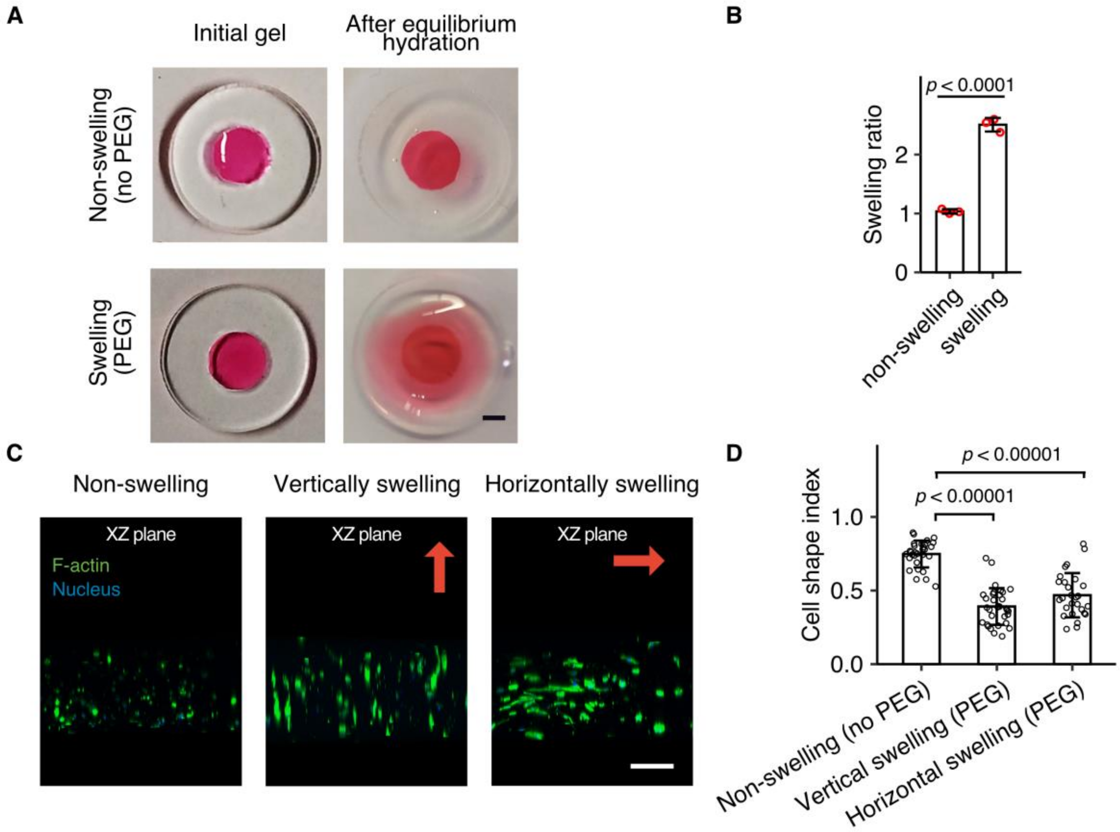

Figure S3. Impact of hydrogel swelling induced by the coupling of hydrophilic PEG molecules to the DexVS backbone. (A), Images illustrate non-swelling and swelling hydrogels before (imaged in air at day 0) and after (imaged in PBS at day 1) equilibrium hydration. Hydrogels functionalized with PEG swell out of the PDMS wells at day 1. *Scale bar*, 2 mm. (B), Hydrogel swelling ratio of the non-swelling versus swelling soft (Young's modulus ~ 0.1 kPa) hydrogels. (n = 3 independent samples). (C), Morphology of hMSCs encapsulated in non-swelling versus swelling soft (Young's modulus ~ 0.1 kPa) hydrogels. The red arrow indicates the swelling direction. Composite fluorescence images showing F-actin (*green*) and nuclei (*blue*) (*scale bar*, 200 μm) (XZ plane shown). (D), Cell shape index of hMSCs encapsulated in the non-swelling versus swelling soft (Young's modulus ~ 0.1 kPa) hydrogels. ($n \geq 25$ cells). All data are presented as a mean ± s.d. Two-tailed unpaired Student's t-test without adjustment was performed for individual comparisons.

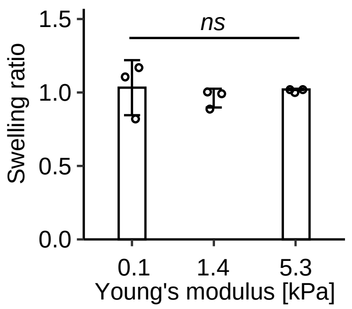

Figure S4. Swelling ratio of DexVS hydrogels crosslinked with 10.1 mM, 25.2 mM and 50.4 mM di-cysteine-HD after equilibration in cell culture medium for 24 hours. Data are presented as a mean ± s.d (n = 3 independent samples). Two-tailed unpaired Student's t-test without adjustment was performed for individual comparisons. *ns*, not significantly different ($p > 0.05$).

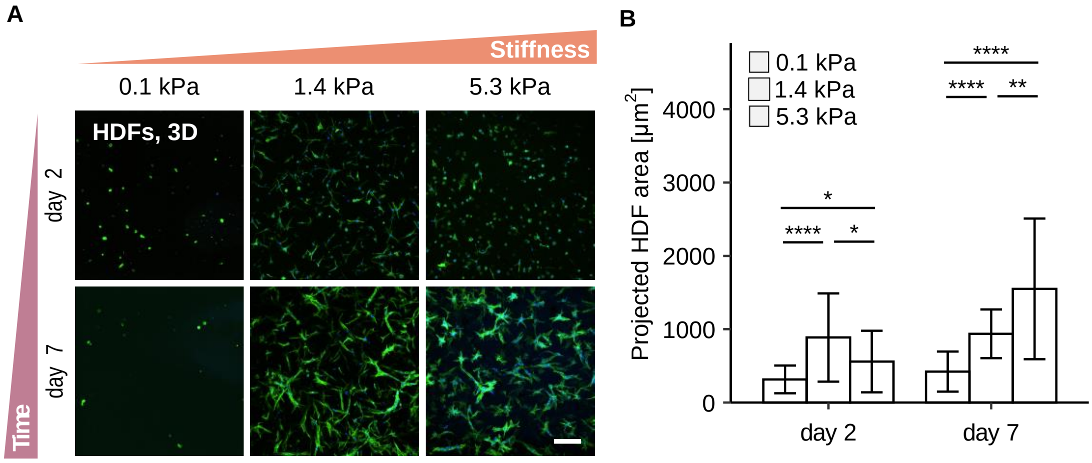

Figure S5. (A), Morphology of HDFs cultured within DexVS hydrogels of varying stiffness for 2 and 7 days. *Scale bar*, 200 µm. Composite fluorescence images showing F-actin (*green*) and nuclei (*blue*). (B), Average cell area of HDFs in (A). $n \geq 20$ cells. All data are presented as a mean ± s.d. Two-tailed unpaired Student's *t*-test without adjustment was performed for individual comparisons. * $p < 0.05$, ** $p < 0.01$, **** $p < 0.0001$.

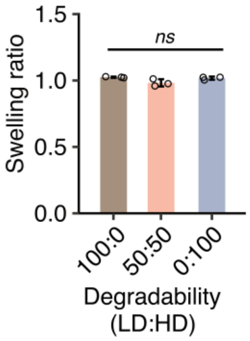

Figure S6. Swelling ratio of DexVS hydrogels crosslinked with mixtures of di-cysteine-LD and di-cysteine-HD peptides after equilibration in cell culture medium for 24 h. The total crosslinker concentration was kept constant at 50.4 mM. (n = 3 independent samples). All data are presented as a mean ± s.d. Two-tailed unpaired Student's t-test without adjustment was performed for individual comparisons. *ns*, not significantly different ($p > 0.05$).

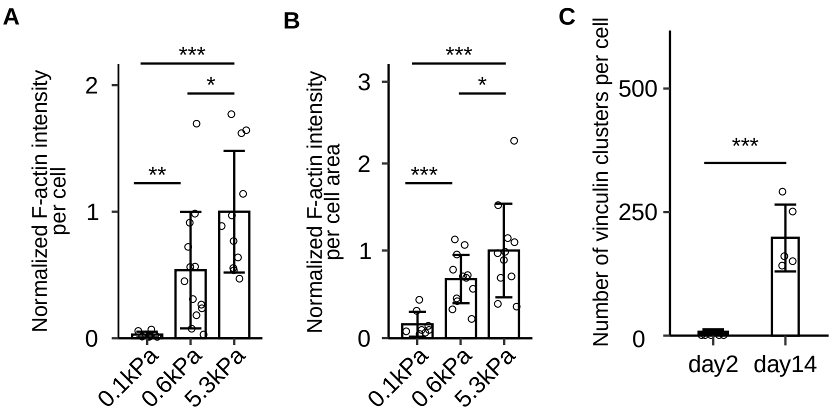

Figure S7. (A, B), Normalized F-actin intensity per cell (A) and per cell area (B) of hMSCs after 14 days of culture in DexVS hydrogels with varying stiffness. $n \geq 10$ cells (C) Number of vinculin clusters per cell of hMSCs cultured in DexVS hydrogels with a Young's modulus of 5.3 kPa at day 2 and day 14. $n = 5$ cells. All data are presented as a mean ± s.d. Two-tailed unpaired Student's *t*-test without adjustment was performed for individual comparisons. * $p < 0.05$, ** $p < 0.001$, *** $p < 0.0001$.
